# Tofacitinib in Leprosy: A Novel Therapeutic Approach in Chronic Recalcitrant Type II Reactions

**DOI:** 10.7759/cureus.74694

**Published:** 2024-11-28

**Authors:** Benaka Suresh, Pooja R Reshmi, Jayalekshmi Jaipal, Gajanan A Pise, Shruthi S Prasad, Naveen Manohar

**Affiliations:** 1 Dermatology, Belagavi Institute of Medical Sciences, Belagavi, IND; 2 Dermatology, Indira Medical College and Hospitals, Pandur, IND; 3 Dermatology, The Oxford Medical College, Hospital, and Research Centre, Bangalore, IND

**Keywords:** erythema nodosum leprosum, lepra reactions, leprosy, thalidomide, tofacitinib

## Abstract

Leprosy is a chronic, infectious, and debilitating disorder that primarily affects the skin and peripheral nerves. The disease course may be complicated by immune-mediated reactions during or after therapy, which may further worsen nerve damage. Type II lepra reaction (T2LR) is a painful inflammatory condition with systemic features, such as fever, tender erythematous nodules, arthritis, neuritis, orchitis, lymphadenitis, and iritis. Erythema nodosum leprosum (ENL), the hallmark of type II lepra reactions, results in hospitalization and consequent impairment in quality of life. The treatment options include long-term high-dose systemic corticosteroids, thalidomide, and/or clofazimine. However, the prognosis is often complicated by the adverse effects of the drugs; therefore, there is a need for alternative and safer therapies.

Herein, we present the case of a 31-year-old male with recurrent lepra reactions who did not respond adequately to steroids. Therefore, we initiated therapy with tofacitinib, a non-selective inhibitor of the Janus kinase/signal transduction and transcription activation (JAK/STAT) pathway. The results included complete resolution of abnormalities on blood laboratory investigations and symptomatic resolution of symptoms. In this article, we delve into the possible role of tofacitinib in T2LR and other inflammatory conditions.

## Introduction

Leprosy is a chronic, infectious, and debilitating disorder caused by the acid-fast bacilli (ACB) *Mycobacterium leprae* (*M. leprae*) and *Mycobacterium lepromatosis* that primarily affects the skin and peripheral nerves. It may be complicated by immune-mediated type I and type II reactions at any time during or after therapy. These reactions may further worsen nerve damage [1].

Type II lepra reaction (T2LR) is a painful inflammatory condition with systemic features, such as fever, tender erythematous nodules, arthritis, neuritis, orchitis, lymphadenitis, and iritis. It affects 5-10% of patients with borderline lepromatous leprosy and 50% of those with lepromatous leprosy [2]. Erythema nodosum leprosum (ENL) is considered the hallmark of type II lepra reactions. ENL results in hospitalization in most cases, and the consequent impairment in quality of life due to ENL is worse than that seen in chronic skin diseases, such as psoriasis [3]. ENL is treated with high doses of systemic corticosteroids, thalidomide, and/or clofazimine for the shortest duration possible. The prognosis is often complicated by the adverse effects of the drugs; therefore, there is a need for alternative and safer therapies [2].

Tofacitinib is a non-selective inhibitor of the Janus kinase/signal transduction and transcription activation (JAK/STAT) pathway. It primarily inhibits JAK1 and JAK3 receptors but also inhibits JAK2 and TYK2 to some extent. Inhibition of JAK1/JAK3 results in lower levels of interferons (IFNs); interleukin (IL)-10 family of cytokines, such as IL-22; and all the gamma-chain cytokines, such as IL-2, IL-4, IL-7, IL-9, IL-15, and IL-21. Of these inflammatory mediators, IL-2, IL-4, IL-15, and IL-7 play a crucial role in the growth and survival of T cells [4].

## Case presentation

A 31-year-old male diagnosed with borderline lepromatous (BL) leprosy one year ago and in the second year of multibacillary-multidrug therapy (MB-MDT) presented with swelling of both feet for 10 days and pain in the knees, ankles, and elbows for one month along with tingling sensations over both hands and feet. Over the course of the disease, he developed multiple episodes of ENL, which were treated with short courses of oral corticosteroids and long-term thalidomide (100 mg TID) and clofazimine (100 mg TID) for over six months. At presentation, he was afebrile with stable vital signs. Physical examination revealed diffuse brownish-red pigmentation of the skin and tenderness of the joints. Nerve examination revealed non-tender grade 2 thickening of the bilateral ulnar and posterior tibial nerves along with decreased sensations of fine and crude touch over both feet; however, motor functions were preserved. Slit-skin smear revealed acid-fast bacilli (AFB) with a bacteriological index (BI) of 2+ and morphological index (MI) of 50%. Laboratory investigations revealed dimorphic anemia (9.2 g/dL) with raised erythrocyte sedimentation rate (45 mm/h) and total leucocyte count (12,710/mm^3^) with neutrophilic leucocytosis. The results of liver and renal function tests were within the normal limits. The patient was on MB-MDT and a tapered dose of clofazimine 100 mg BD as part of the treatment of a previous episode of ENL. Ten days after admission, he developed a fever (101 °F) with aggravated joint pain and tingling sensations over the extremities. Laboratory investigations revealed elevated serum levels of C-reactive protein (CRP) (116 mg/dL) and alkaline phosphatase (ALP) (175.8 U/L) with leucocytosis (12,900/mm^3^). He was diagnosed with arthritis-predominant type 2 lepra reaction. Additionally, other causes of fever were ruled out based on dengue NS1, IgM, and IgG test, Widal test, blood smear for malaria, and sputum cartridge-based nucleic acid amplification test (CB-NAAT) for tuberculosis. The patient was comprehensively worked up with chest X-ray, liver and renal function tests, lipid and urine analysis, and initiated on oral extended-release tofacitinib 11 mg daily. 

A week later, the patient reported improvements in fever, arthritis, and tingling sensation over the extremities. Serum C-reactive protein (CRP) level dropped to 54.2 mg/dL, and total leucocyte count reduced to 9000/mm3 with neutrophils constituting 70% of the value. A month later, serum CRP level was reduced to 0.2 mg/dL (Table 1).

**Table 1:** Changes in serum haematological and biochemical parameters following oral daily tofacitinib therapy with extended-release 11 mg formulation Parameters with meaningful changes are highlighted in bold.

Parameter	At admission	Day 1	Day 7	Day 30	Reference range
Haemoglobin (g%)	9.2	8.9	9.1	9.1	12.5–17.5
Total leucocyte count (/mm^3^)	12,710	12,900	9000	10,500	4,500–10,000
Neutrophils (%)	83	84	70	68	55-70
Lymphocytes (%)	12	13	25	22	20-40
Basophils (%)	0	0	0	00	0.5-1
Monocytes (%)	2	1	2	02	2-8
Platelet count (/mm^3^)	450,000	500,000	520,000	340,000	150,000-350,000
C-reactive protein (mg/dL)	118	116	54.2	0.2	<1
Liver Function Tests					
Total bilirubin (mg/dL)	0.8	0.4	0.3	0.2	0.3-1.0
Direct bilirubin (mg/dL)	0.3	0.2	0.1	0.1	0.1-0.3
Indirect bilirubin (mg/dL)	0.5	0.2	0.2	0.1	0.2-0.8
Globulin (g/dL)	3.0	2.0	3.0	2.9	2.0-3.5
Alkaline phosphatase (U/L)	91	175.8	137	105.9	4-40
Renal function tests					
Urea (mg/dL)	19.8	20.4	18.3	19.5	8-24
Creatinine (mg/dL)	1.1	1.2	1.1	1.2	0.2-1.2

## Discussion

In this study, we evaluated the role of oral tofacitinib in a patient with T2LR who could not be administered the standard therapy of corticosteroids in view of leucocytosis and anemia. The role of tofacitinib in T2LR has not been reported yet; additionally, its mechanism of action in resolving inflammation has not been completely elucidated. To the best of our knowledge, this is the first report on the use of tofacitinib in T2LR. A literature search on clinicaltrials.gov showed no current studies on the use of tofacitinib in T2LR as of 31/10/2024.

Pathogenesis of type II lepra reaction and tofacitinib

Neutrophils

Neutrophils are the hallmark of ENL and are seen in early lesions of ENL. Lee et al. demonstrated that the activation of TLR2/Fc increases IL-1β, which, along with IFN-γ, induces E-selectin expression in endothelial cells. This, in turn, leads to the adhesion of neutrophils to endothelial cells and the initiation of inflammation in ENL [5]. Additionally, tofacitinib inhibits JAK-dependent IL-6 signaling pathway, which is responsible for neutrophil activation. We believe that these pathways are involved in the inhibition of neutrophils by tofacitinib. Interestingly, the fall in neutrophil count appears to be reversible as well as dose-dependent [6].

Cytokines

**Figure 1:**
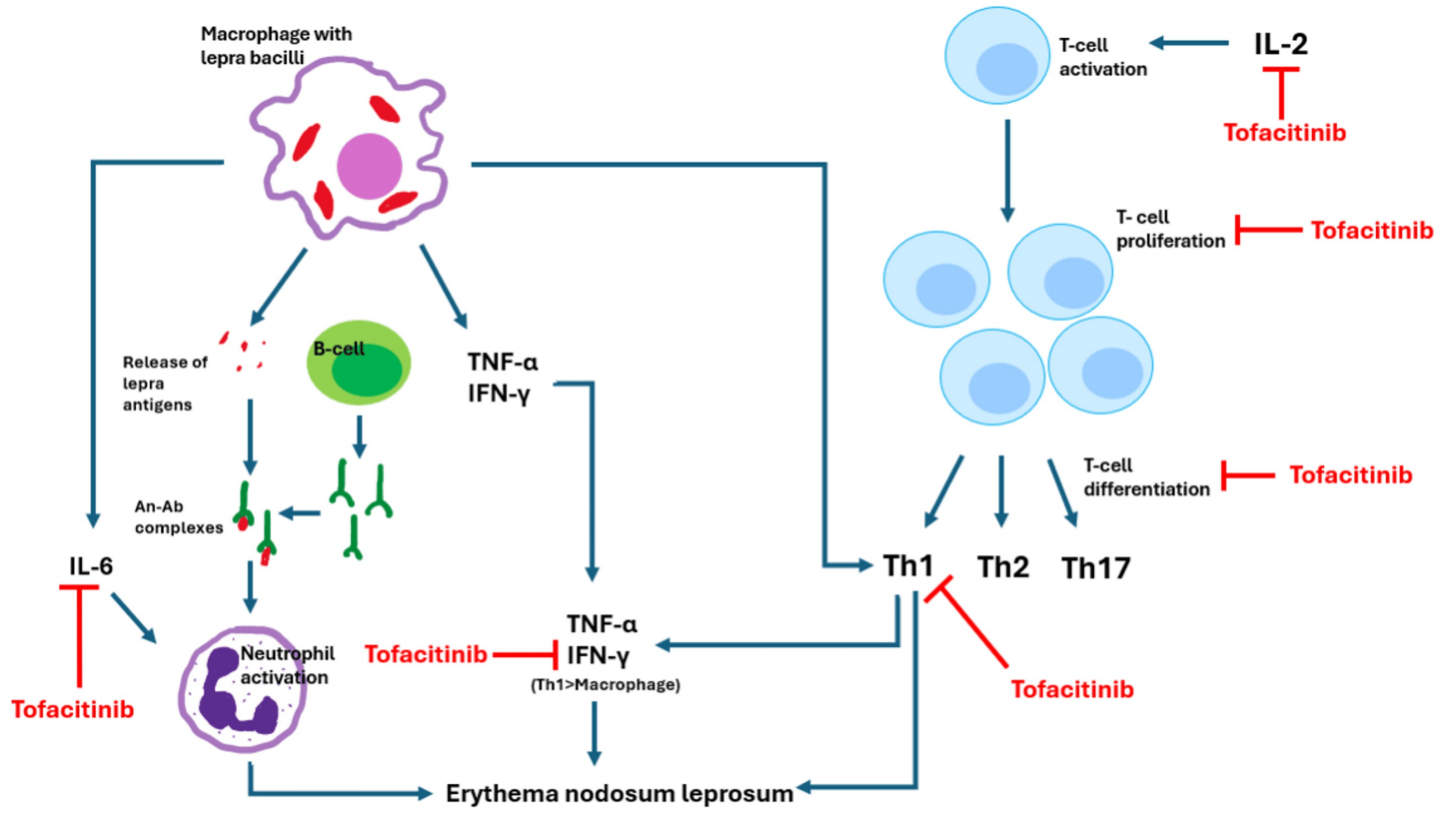
Mechanisms of action of tofacitinib in type II lepra reaction. Tofacitinib acts at multiple points in the pathogenesis of type II lepra reaction. Illustration by: Benaka Suresh

TNF-α: There is an abundance of literature on the role of TNF-α in T2LR [7-9]; however, the inferences remain debatable. Most studies have reported elevated serum levels of TNF-α in T2LR, thus highlighting TNF-α as the predominant inflammatory cytokine in T2LR [9]. TNF-α in ENL is primarily produced by monocytes, such as Th1 cells and macrophages [8]. Tofacitinib is a potent inhibitor of γc-cytokine signaling in mice models as it blocks IL-2-driven T-cell proliferation [10]. Therefore, it can be postulated that tofacitinib indirectly inhibits TNF-α by modulating T-cell proliferation (Figure 1).

IFN-γ: Studies have consistently reported elevated serum levels of IFN-γ in T2LR relative to the serum levels of TNF-α. Sampaio et al. reported that IFN-γ-induced ENL was observed in most of the patients after 7 months of multiple intradermal injections of IFN-γ along with MB-MDT [11]. Some of the actions of tofacitinib include inhibition of IFN-γ, inhibition of the release of IL-17 from CD4+ T-cells, and lowering mRNA levels of IFN-γ and IL-17 in a dose-dependent manner [12].

However, whether tofacitinib-induced inhibition of IFN- γ plays a crucial role in T2LR remains unclear.

T-cells

In a study on the role of T-cells in T2LR, it was proposed that spontaneous activation of T-cells is the primary event that leads to the activation of macrophages [13]. Subsequently, the activation of macrophages leads to the following three events: 1) processing of *M. leprae* and the release of the processed antigens; 2) antigen presentation to Th1 cells; and 3) the production of proinflammatory cytokines, such as IFN-γ, TNF-α, and other similar molecules.

Antigen presentation to Th1 cells results in the release of chemokines, recruitment of macrophages, and further release of pro-inflammatory cytokines, such as IFN-γ and TNF-α, which increase the levels of vascular adhesion molecules.

The processed antigen molecules combine with preformed antibodies to form an antigen-antibody complex, which combines with C1q, thus ultimately resulting in a protein complex and the recruitment of neutrophils to the site. These pro-inflammatory cytokines along with Th17 and Th1 cells and immune complexes lead to tissue damage [13].

Ghoreschi et al. reported that tofacitinib not only inhibits the major proinflammatory cytokines involved in the initial stages of T2LR, such as IFN-γ and TNF-α, but it also inhibits the differentiation of T-cells (Th1 and Th2) and Th17 cells. It was also noted that tofacitinib has only a modest action on IL-12-induced STAT4 activation but has a profound inhibitory effect on STAT1 activation in T-cells induced by either IL-12 or IFN-γ. The authors suggested that the inhibition of IFN-γ signaling alone is likely responsible for Th1 suppression [10].

We acknowledge a few limitations of this report. The patient was treated in multiple hospitals and leprosy centers, which limited the temporal data collection for serial monitoring. Additionally, due to the case report design of the report, we could not correlate clinical changes with mycobacterial changes over time. We hope to overcome these points in a future longitudinal study with a bigger sample size. 

## Conclusions

We report a potential novel therapeutic utility of tofacitinib in T2LR, where it not only blocks TNF-α, but also acts on several inflammatory cells and cytokines. An additional advantage of tofacitinib is that the cost of therapy is approximately a third of the therapy with thalidomide. This case report may serve as the foundation for further studies regarding the utility of tofacitinib in acute inflammatory conditions, such as ENL. Larger, well-designed studies and randomized control trials are warranted to further explore use of tofacitinib in these conditions. 
